# Ferroptosis Inducers Combined with Copper Ionophores Aggravate Lung Cancer-Related Fatigue via GSH Depletion and FKBP5-Associated Impairment of Nrf2/HO-1 Signaling

**DOI:** 10.3390/cells15151394

**Published:** 2026-07-31

**Authors:** Ming Chen, Ying Pang, Yi He, Yunan Ma, Lili Tang

**Affiliations:** 1 Key Laboratory of Carcinogenesis and Translational Research (Ministry of Education/Beijing), Department of Psycho-Oncology, Peking University Cancer Hospital & Institute, Beijing 100142, China; 2311110700@stu.pku.edu.cn (M.C.); pangying@pku.edu.cn (Y.P.); heyi@pku.edu.cn (Y.H.); 2 Key Laboratory of Carcinogenesis and Translational Research (Ministry of Education/Beijing), Department of Laboratory Animal, Peking University Cancer Hospital & Institute, Beijing 100142, China; yunanma0324@bjcancer.org.cn

**Keywords:** cancer-related fatigue, cuproptosis, ferroptosis, copper ionophores, ferroptosis inducers

## Abstract

**Highlights:**

**What are the main findings?**

- Ferroptosis inducers (FINs) sensitize skeletal muscle cells to copper ionophore (CIN)–Cu stress, enhancing cuproptosis hallmarks such as DLAT aggregation, mitochondrial dysfunction, ROS accumulation, and cytotoxicity.
- FINs deplete intracellular glutathione (GSH) and induce FKBP5 upregulation, which is associated with impaired Nrf2/HO-1 antioxidant signaling and increased vulnerability to cuproptosis.

**What are the implications of the main findings?**

- Copper chelation with tetrathiomolybdate (TTM) counteracts FIN–CIN–Cu synergy, supporting a copper-dependent mechanism underlying aggravated skeletal muscle injury.
- In an orthotopic lung cancer mouse model, CIN–Cu + FINs worsen fatigue-like behaviors, whereas TTM or molecular hydrogen partially improves performance, suggesting potential supportive intervention strategies.

**Abstract:**

Cancer-related fatigue (CRF) remains difficult to manage, and the impact of metal ion-regulated cell death on peripheral fatigue during anticancer therapy is unclear. Here, we investigated whether ferroptosis inducers (FINs) potentiate copper ionophore (CIN)-triggered cuproptosis in skeletal muscle and aggravate lung cancer-related fatigue (LCaRF), and evaluated redox-based interventions. LCaRF cellular models were established using C2C12 exposed to LLC/M109 tumor-conditioned supernatants and treated with FINs (sorafenib/erastin) plus CIN + CuCl_2_ (CIN–Cu + FINs). Cell viability, lipid peroxidation, DLAT aggregation (cuproptosis hallmark), copper/glutathione (GSH), mitochondrial function, and FKBP5/Nrf2–HO-1 signaling were assessed with pharmacologic and genetic modulation. An orthotopic lung cancer mouse model underwent wheel-running, tail suspension, and open-field testing with tetrathiomolybdate (TTM) or hydrogen as interventions. FINs sensitized C2C12 cells to CIN–Cu cytotoxicity and increased DLAT aggregation; copper chelation with TTM attenuated these effects. FINs depleted GSH and amplified mitochondrial dysfunction/ROS; exogenous GSH or hydrogen reduced DLAT aggregation and restored mitochondrial indices. FKBP5 was markedly upregulated by CIN–Cu + FINs and linked to suppressed antioxidant defense (Nrf2/HO-1). In vivo, CIN–Cu + FIN treatment exacerbated fatigue-like behaviors, while TTM or hydrogen partially improved performance. FIN–CIN combinations may aggravate skeletal muscle injury and fatigue-like phenotypes in LCaRF models by promoting cuproptosis via GSH depletion and FKBP5/Nrf2-HO-1 dysregulation.

## 1. Introduction

Cancer-related fatigue (CRF) is a common and debilitating symptom throughout the cancer trajectory. Meta-analyses indicate that approximately 70–80% of patients experience clinically significant fatigue during or after treatment, with many survivors developing severe fatigue [1]. CRF substantially impairs health-related quality of life, employment, and daily functioning, imposing considerable psychosocial and economic burdens on patients, families, and society [2]. Current pharmacological and non-pharmacological interventions have limited efficacy; psychostimulants, antidepressants, corticosteroids, and other agents show inconsistent benefits and are often restricted by adverse effects or narrow indications [3]. Therefore, elucidating the biological basis of CRF is essential for developing mechanism-based therapies.

CRF has been linked to systemic inflammation, neuroendocrine disruption, mitochondrial dysfunction, anemia, and psychosocial factors [4,5]. However, its complexity suggests that additional pathways may contribute to central and peripheral fatigue. Metal ion homeostasis is critical for cellular function, particularly in mitochondria, which regulate energy production and redox balance in high-demand tissues such as skeletal muscle and brain [6,7]. Ferroptosis is an iron-dependent regulated cell death characterized by lipid peroxide accumulation and plasma membrane damage [8]. Ferroptosis inducers (FINs) have shown therapeutic potential in ferroptosis-sensitive cancers and in overcoming resistance to radiotherapy, chemotherapy, and immunotherapy [9,10,11]. For example, targeting *CPT1A* induces ferroptosis by suppressing intracellular glutathione (GSH) synthesis and inhibiting *GPX4*-mediated antioxidant defense, showing efficacy in preclinical cancer models and lung cancer immunotherapy [12]. Cuproptosis is a copper-triggered cell death process driven by mitochondrial copper overload, aggregation of lipoylated TCA-cycle proteins, destabilization of iron–sulfur cluster proteins, and proteotoxic stress [13]. A PROTAC-based copper accumulation sensitizer has recently shown favorable therapeutic effects in lung cancer [14]. Although ferroptosis and cuproptosis are increasingly recognized in oncology, their roles in CRF remain poorly defined.

FK506-binding protein 51, encoded by *FKBP5*, is a stress-responsive immunophilin that regulates glucocorticoid receptor signaling [15]. Beyond endocrine functions, FKBP5 also regulates redox homeostasis and mitochondrial integrity [16,17]. *Fkbp5* overexpression reversed the effects of PRRX1 silencing on excessive mitophagy and cardiomyocyte ferroptosis in OGD/R-treated AC16 cells [18]. Although the relationship between FKBP5 and cuproptosis remains unclear, copper ionophores (CINs) can increase oxidative stress and impair mitochondrial function [19], suggesting that FKBP5 may participate in cuproptosis regulation and contribute to CRF driven by ion-homeostasis imbalance.

Here, using a lung cancer-related fatigue (LCaRF) model, we found that FINs, including sorafenib and erastin, enhanced CIN-induced cuproptosis in skeletal muscle and aggravated peripheral fatigue-like phenotypes. Mechanistically, CIN–Cu + FIN exposure induced GSH depletion and oxidative stress, together with *Fkbp5* upregulation and impaired Nrf2/HO-1 antioxidant signaling, which collectively promoted mitochondrial dysfunction, DLAT aggregation, and cuproptosis-related skeletal muscle injury. Moreover, FIN- and CIN–Cu-induced LCaRF was alleviated by the redox agent hydrogen.

## 2. Materials and Methods

### 2.1. Cells, Antibodies and Chemicals

The C2C12, LLC and M109 cell lines were procured from the Chinese Academy of Medical Sciences (Beijing, China). All cell lines were cultured in Dulbecco’s modified Eagle medium (DMEM; Servicebio, Wuhan, China) supplemented with 10% fetal bovine serum (FBS) (FBS; Gibco, Grand Island, NY, USA) and 100 U/mL penicillin-streptomycin (P/S) at 37 °C with 5% CO_2_. Media were then substituted with DMEM supplemented with 2% horse serum (Gibco) and grown for three days for myotube induction. C2C12 cells following differentiation induction were stained with Giemsa stain (Beyotime, Shanghai, China; Cat#C0131) according to the manufacturer’s instructions for morphological observation. Mouse C2C12 myoblasts were cultured in proliferation medium containing DMEM, 10% FBS, and 1% penicillin/streptomycin until approximately 70% confluence was reached. Differentiation was induced using DMEM supplemented with 2% horse serum and 1% penicillin/streptomycin, with the medium replaced every day. After 3 days of differentiation, C2C12 myotubes were placed in the lower chamber and co-cultured with LLC or M109 cells seeded in the upper Transwell inserts to establish an LCaRF cell model. This indirect co-culture system enabled tumor cell-derived paracrine factors to act on differentiated C2C12 myotubes while preventing direct contact between the two cell types [20,21,22]. Subsequently, drug treatment was administered for 24 h to LCaRF cell model [23]. The names and concentrations of the drugs used are as follows: 2.5 μM sorafenib (Sora), 2.5 μM erastin (Era), 5 μM tetrathiomolybdate (TTM), 2.5 mM tetraethylenepentamine (TEPA), 5 mM glutathione (GSH), 5 μM Tertiary butylhydroquinone (TBHQ), or 1 μM ML385. Depending on the different cell supernatants added to the LCaRF model, we used different CINs. For LCaRF model with LLC cell line supernatant added, we used 5 nM Elesclomol (Els) + 1 μM CuCl_2_; while for LCaRF model with M109 cell line supernatant added, we used 50 nM diethyldithiocarbamate (DDC) + 1 μM CuCl_2_. The sources of the antibodies and reagents employed in this study are provided in Table S1.

### 2.2. RNA Extraction and Quantitative Real-Time Polymerase Chain Reaction (qRT-PCR)

Total RNA was extracted using TRIzol Universal Reagent (Invitrogen, Carlsbad, CA, USA) and subsequently reverse transcribed with the PrimeScript™ RT Reagent Kit (Yeasen, Shanghai, China). qRT-PCR was performed using 2X Universal SYBR Green Fast qPCR Mix (Abclonal, Wuhan, China; Cat#RK21203). Gene levels were evaluated through the 2^−ΔΔCT^ method. Supplementary Table S2 lists the primer sequences used in this study.

### 2.3. Western Blotting (WB)

Cell samples were washed with precooled PBS and lysed in RIPA lysis buffer. Lysates were incubated on ice for 30 min, then centrifuged at 12,000× *g* for 10 min, and supernatants were collected. Protein concentration was determined using the BCA assay kit (Beyotime, Shanghai, China; Cat#P0010S). Total protein was mixed with sample buffer, denatured at 100 °C for 10 min, and separated by SDS-PAGE. Proteins were transferred to PVDF membranes (Millipore, Burlington, MA, USA; Cat# ISEQ00010), blocked with 5% skim milk to prevent nonspecific binding, and incubated overnight at 4 °C with primary antibodies. After washing with PBST (0.5% Tween), membranes were incubated with secondary antibodies at 25 °C for 1 h, followed by three washes with PBST for signal detection. Western blot band intensities were quantified using ImageJ (v1.53t). After background subtraction, each target protein signal was normalized to the corresponding GAPDH signal from the same lane. The resulting Target/GAPDH densitometric ratios were used for quantification. Quantification was performed based on independent biological replicates.

### 2.4. Co-Immunoprecipitation Assay

Co-immunoprecipitation (Co-IP) was performed to examine the potential association between FKBP5 and Nrf2. Briefly, C2C12 cells in the LCaRF model were lysed using ice-cold IP lysis buffer supplemented with protease and phosphatase inhibitors. Equal amounts of total protein were incubated overnight at 4 °C with anti-FKBP5 antibody or control IgG, followed by incubation with protein A/G magnetic beads. The immunoprecipitates were washed and eluted with SDS loading buffer, and the eluted proteins were analyzed by Western blotting using antibodies against Nrf2 and FKBP5. Whole-cell lysates were used as input controls.

### 2.5. RNA Interference

Gene overexpression vectors were generated by inserting their sequences into the pcDNA 3.1 vectors. The vectors or siRNAs mentioned in this article were synthesized by GenePharma Company (Shanghai, China) and transfected into cells with Lipofectamine™ RNAiMAX transfection reagent at a final concentration of 20–50 nM. The sequences of the siRNA employed in this study are presented in Table S3.

### 2.6. RNA Sequencing and Bioinformatics Analysis

The RNA extraction and sequencing methods used were identical to those in a previous study [24,25]. Total RNA was extracted from the Control group (*n* = 3) and Els–Cu + Sora group (5 nM Els + 2.5 μM Sora + 1 μM CuCl_2_; *n* = 3) in the LCaRF model using TRIzol reagent according to the manufacturer’s protocol. RNA quality was assessed using an Agilent 2100 Bioanalyzer (Agilent Technologies, Santa Clara, CA, USA), and RNA-seq libraries were constructed by CapitalBio Technology (Beijing, China). Ribosomal RNA was removed using the Ribo-Zero Magnetic Kit (Epicentre, Madison, WI, USA; Cat#MRZB12424), followed by RNA fragmentation, library preparation, quantification, and paired-end sequencing on an Illumina HiSeq platform. Raw sequencing reads were quality-checked using FastQC (v0.11.5), filtered with NGSQC (v2.3.3), and aligned to the mouse genome (GRCm38/mm10) using HISAT2 (v2.1.0). Gene and transcript assembly and quantification were performed using StringTie (v1.3.3b). Sample correlation and gene annotation analyses were conducted using BioMAS v3.0. Differentially expressed transcripts were identified in R (v4.2.3) using thresholds of |log fold change| > 1.25 and *p* < 0.05, followed by GO and KEGG enrichment analyses to explore biological functions and pathways.

The screening process for candidate CRF diagnostic marker genes was based on our previous research [26]. GSE14786 and GSE30174 were analyzed independently because they represent distinct CRF cohorts with different cancer types, sex backgrounds, treatment contexts, and sample sources. The two datasets were not merged for differential expression analysis. Instead, within-dataset analyses were performed separately, and the results were compared across cohorts to evaluate whether candidate CRF-associated genes showed consistent relevance across different cancer-related fatigue contexts. Correlation analysis was performed using the ggplot2 package in R software (v4.2.3) with the Spearman method. Weight analysis was carried out with the utilization of the survey and cluster packages in R software (v4.2.3). Sensitivity analysis of candidate CRF diagnostic marker genes and *COX5B* across multiple tumor cell lines was conducted using data from the DepMap database [27].

### 2.7. Lipid Peroxidation Analysis

The experiments were conducted as previously described. After being treated with the indicated ferroptosis inducers, cells were incubated with serum-free medium containing 5 µM BODIPY-C11 for 30 min at 37 °C. After being washed three times with 1× PBS, the cells were collected and resuspended in 1× PBS for subsequent flow cytometric analysis. The oxidation of the polyunsaturated butadienyl moiety of the dye results in a shift in the fluorescence emission peak from ~590 nm to ~510 nm.

### 2.8. GSH Assay

The relative GSH concentration in cell lysates was assessed using a GSH Assay Kit (Beyotime, Shanghai, China; Cat#S0053) according to the manufacturer’s instructions. Simply, cell sediments were collected and treated with protein clear solution. Finally, the GSH level was calculated by measuring the OD value at 412 nm after mixing with NADPH solution.

### 2.9. Cell Growth Assay

Cell growth assay was applied with the CCK-8 kit (Servicebio, Wuhan, China; Cat#G4103). Briefly, cells were seeded into a 96-well plate overnight and treated as indicated, and CCK-8 reagents were added to each well, after which the plates were placed in a humidity incubator (37 °C, 5% CO_2_). The absorbance measurements were carried out at 450 nm using a Synergy microplate reader (BioTek Instruments, Winooski, VT, USA). Samples were prepared at least in triplicate.

### 2.10. Propidium Iodide (PI) Staining

The logarithmic growth phase cells were digested and seeded evenly in 24-well plates (80,000 cells per well) and cultured for 24 h. The well-attached cells were lysed as previously described. Subsequently, 10 μg/mL Hoechst 33342/PI double-staining reagent (EallBio, Beijing, China; Cat#02.10553) was added and incubated at room temperature in the dark for 30 min, followed by imaging using a fluorescence microscope (Nikon, Tokyo, Japan).

### 2.11. Mitochondrial Respiratory Chain Complex (MRCC) IV Activity

MRCC IV activity was measured using a micro assay kit (Abbkine, Wuhan, China; Cat#KTB1880) according to the manufacturer’s protocol. C2C12 cells were lysed at 4 °C and centrifuged at 600× *g* for 10 min, then at 11,000× *g* for 10 min, both at 4 °C, to isolate mitochondria. Isolated mitochondria were resuspended in assay buffer, and reagents and samples were added to a UV plate as directed. Absorbance was recorded at baseline and every 2 min at 550 nm using a Synergy microplate reader (Bio-Tek Instruments). Enzyme activity was calculated from the rate of absorbance change and normalized to protein content determined by the BCA Protein Assay Kit.

### 2.12. Immunofluorescence (IF) Assay

Cells were seeded on coverslips in 6-well plates overnight and treated as indicated. After fixation with 4% paraformaldehyde for 10 min, cells were permeabilized with 0.25% Triton X-100 for 10 min, blocked with 3% BSA for 1 h, and incubated overnight at 4 °C with primary antibodies against DLAT, COX5B, or FDX1 (1:500). After washing with 0.1% PBS-T, cells were incubated with secondary antibodies at 37 °C for 1 h, counterstained and mounted with DAPI-containing medium. For mitochondrial staining, cells were incubated with a 200 nM mitochondrial red fluorescent probe for 30 min before fixation. For ROS detection, cells were loaded with the fluorescent probe in serum-free medium for 45 min according to the manufacturer’s protocol, then fixed and imaged. For tissue immunofluorescence, gastrocnemius muscle samples were fixed in 4% paraformaldehyde, paraffin-embedded, sectioned at 10 μm, and processed for staining.

### 2.13. Copper Measurements

Copper levels in cells and muscle tissues were measured using a copper assay kit (Sigma-Aldrich, St. Louis, MO, USA; Cat#MAK127) as previously described. Muscle samples were homogenized in sodium phosphate buffer (pH 7.5), while C2C12 cells from different groups were collected and homogenized in distilled water. Assay reagents were added according to the manufacturer’s protocol, and absorbance was measured at 359 nm using a microplate reader. Protein concentrations were determined with a BCA Protein Assay Kit to normalize copper content.

### 2.14. Hydrogen-Rich Medium Preparation

Hydrogen-rich medium was produced by injecting gas using the hydrogen generator (Shanghai Asclepius Medical Technology Co., Ltd., Shanghai, China; Cat#AMS-H-03/30C) into DMEM medium for 10 min, and the hydrogen dissolved in the reaction solution. The needle-type hydrogen sensor (Unisense, Aarhus N, Denmark) showed that hydrogen saturation in the culture medium was achieved within 10 min of gas supplementation (2 L/min), with hydrogen concentration reaching 0.85 mM (Figure S3C). To measure the hydrogen concentration in the reaction system, the sensor was inserted below the liquid surface.

### 2.15. Mice Model

All animal experiments were performed in accordance with the Institutional Animal Care and Use Committee guidelines of the Beijing Cancer Hospital (Ethics Approval Number: EAEC 2025-10). C57BL/6J mice (8–9 weeks old, weighing 20–24 g) were obtained from Beijing Vital River Laboratory Animal Technology (China). Mice were housed under specific pathogen-free (SPF) conditions in ventilated cages with a controlled 12 h light/dark cycle, temperature, and humidity, with ad libitum access to enriched food and water. Luciferase-expressing LLC cells were resuspended in PBS, and 5  ×  10^5^ of cells were administered to each mouse via tracheal instillation [28]. Maintain an upright position for 15 min after intrapulmonary administration to ensure complete delivery of the LLC cells to the lungs. The voluntary running wheel and tail suspension tests were performed on the third day after model establishment. Mice were assigned to a non-tumor control group (Con) and five LCa-bearing groups: vehicle, elesclomol (40 mg/kg) + CuCl_2_ (0.06 mg/kg), sorafenib (10 mg/kg)  +  elesclomol (40 mg/kg) + CuCl_2_ (0.06 mg/kg), sorafenib (10 mg/kg)  +  elesclomol (40 mg/kg) + CuCl_2_ (0.06 mg/kg) + TTM (10 mg/kg), sorafenib (10 mg/kg)  +  elesclomol (40 mg/kg) + CuCl_2_ (0.06 mg/kg) + hydrogen (2 L/min). Hydrogen was administered via closed-space ventilation for 30 min as an intervention, whereas other drugs were delivered by intraperitoneal injection, with five administrations per week.

### 2.16. Animal Fatigue Behavioral Assessment

Fatigue-like behaviors in mice were assessed using voluntary wheel running activity, the open field test (OFT), and the tail suspension test (TST). For voluntary wheel running, mice were individually housed in cages equipped with a wireless running wheel with a diameter of 15 cm (Tafit, Hangzhou, China), as previously described [29]. Wheel revolutions, activity time, and running distance were recorded overnight for 12 h before and during the experiment twice per week. For the OFT, mice were placed in a square chamber measuring 50 cm × 50 cm × 40 cm for 5 min to assess fatigue-like behavior [30]. Total distance traveled, global activity in the center, and time spent in the border area were recorded and analyzed using Smart 3.5 software. After each trial, the apparatus was cleaned with rubbing alcohol to eliminate residual odors. For the TST, mice were suspended approximately 50 cm above the floor by adhesive tape placed 1 cm from the tip of the tail, as previously reported for evaluating cancer-related fatigue-like behavior [31,32]. Immobility time was recorded for 6 min per mouse, and the last 4 min were used for subsequent analysis.

### 2.17. Transmission Electron Microscopy (TEM) Analysis

For TEM analysis, the 1 mm^3^ specimens derived from the gastrocnemius muscle tissues were prepared according to established methods [33]. Tissue samples were fixed, dehydrated, and sliced into 70 nm sections. The sections were mounted on grids and stained with lead citrate and uranyl acetate, then examined under a transmission electron microscope (Thermo Fisher Scientific, Hillsboro, OR, USA).

### 2.18. Bioluminescence Imaging

D-luciferin (30 mg/mL, 100 μL) was injected into mice 15 min before BLI. Mice were anesthetized with 2% isoflurane and 0.3 L/min oxygen. Images were acquired and analyzed using Live Image 4.5 software. For quantification, tumor ROIs were defined using white light images, and muscle ROIs of similar size and opposite position to the tumor were selected as controls. Background signal was subtracted from each luminescence image, and the average fluorescence signal within each ROI was calculated.

### 2.19. Quantification of Immunofluorescence Images

Immunofluorescence images were quantified usingImageJ (v1.53t)under identical thresholding parameters for all groups within each experiment. DLAT aggregation was quantified as the total aggregated DLAT-positive area normalized to the number of DAPI-positive nuclei in the same field and expressed as the DLAT aggregation index. Nrf2 nuclear translocation was quantified as the nuclear/cytoplasmic fluorescence intensity ratio based on DAPI-defined nuclear regions and corresponding cytoplasmic regions. FDX1, FKBP5, COX5B, and Nrf2 fluorescence intensities were quantified as background-corrected mean fluorescence intensity and expressed relative to the corresponding control group where applicable. Quantification was performed from at least three independent experiments. For each group, randomly selected microscopic fields were analyzed, and all clearly identifiable DAPI-positive nuclei or cells within the selected fields were included where applicable.

### 2.20. Statistical Analysis

Statistical analyses were performed using GraphPad Prism 8 software. Data are presented as mean ± SD. For cell-based experiments, n refers to independent biological replicates, and technical replicates were not treated as independent biological replicates. Unless otherwise indicated, cell-based experiments were performed with at least three independent biological replicates. For animal experiments, each mouse was considered one biological replicate. Longitudinal body weight and behavioral measurements were analyzed using mixed-effects analysis with treatment group and time as factors and individual mouse identity as the repeated-measures factor, followed by Šídák-adjusted post hoc comparisons at individual time points. Other grouped or dose–response datasets were analyzed using one-way or two-way ANOVA followed by appropriate multiple-comparison tests. Two-group comparisons were performed using two-tailed *t*-tests or Wilcoxon rank-sum tests as appropriate. Non-parametric tests were used when data did not meet parametric assumptions. A *p*-value < 0.05 was considered statistically significant.

## 3. Results

### 3.1. FINs Promoted Copper-Induced Cell Death in the LCaRF Models

**Figure 1:**
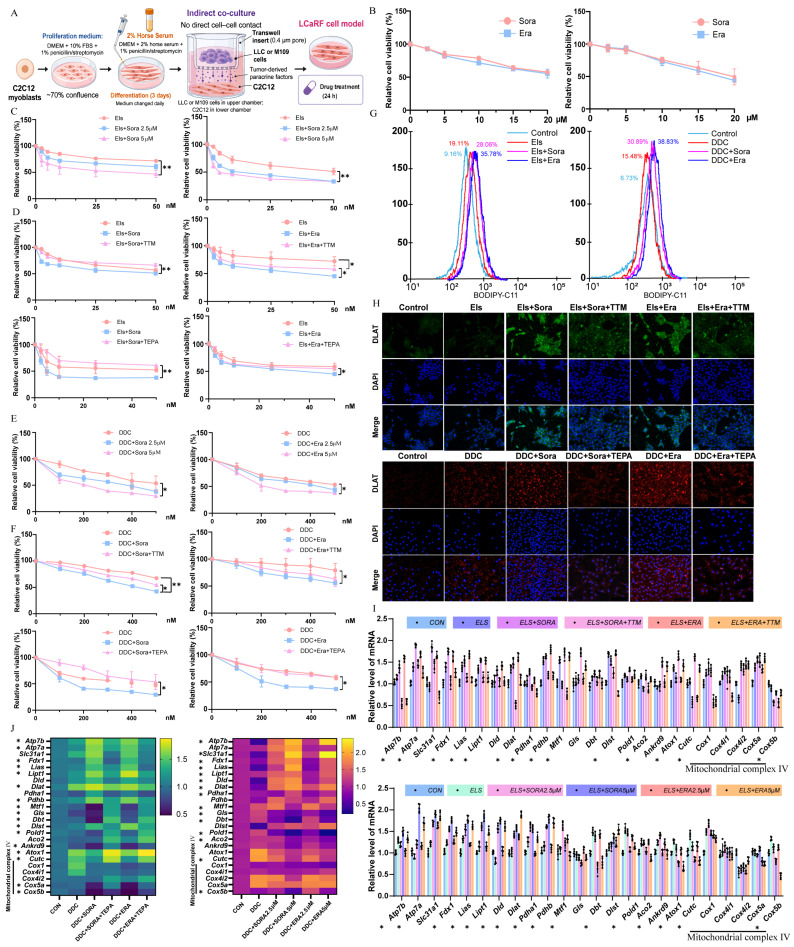
Ferroptosis inducers (FINs) enhanced copper ionophore (CIN)-induced cell death. (**A**). Flowchart of the lung cancer-related fatigue (LCaRF) cell model induction procedure. (**B**). C2C12 cell viability after 24 h treatment with the indicated concentrations of Sora or Era was measured using CCK-8 assays. LLC (left panel) or M109 (right panel) supernatants were used in a LCaRF cell model. (**C**,**E**). Cell viability of C2C12 cells after Elesclomol (Els 24 h) or diethyldithiocarbamate (DDC 24 h) treatment with Sora (0, 2.5, 5 μM) or Era (0, 2.5, 5 μM) was measured with a CCK-8 assay. (**D**,**F**). Cell viability of C2C12 cells after Els or DDC 24 h treatment, together with DMSO, 2.5 μM Sora (or 2.5 μM Era), 2.5 μM Sora (or 2.5 μM Era) + 5 μM tetrathiomolybdate (TTM) or 2.5 μM Sora (or 2.5 μM Era) + 2.5 mM tetraethylenepentamine (TEPA) was measured with a CCK-8 assay. (**G**). Lipid peroxides of C2C12 cells after Els or DDC 24 h treatment, together with DMSO, 2.5 μM Sora or 2.5 μM Era for 24 h, were detected with BODIPY-C11. (**H**). C2C12 cells were treated with indicated drugs for 24 h, DLAT protein aggregation was analyzed by immunofluorescence (IF) (red or green, DLAT; blue, DAPI). Scale bar: 100 μm. (**I**,**J**). After treating C2C12 cells with indicated drugs, qRT-PCR was performed to determine the mRNA expression. For panels (**C**–**J**), media were supplemented with 1 µM CuCl_2_. Data are presented as mean ± SD from three independent biological experiments unless otherwise indicated. Indicated comparisons are shown in the corresponding panels. * *p* < 0.05, ** *p* < 0.01.

The LCaRF cell model was established as previously described, and the C2C12 induction workflow is shown in Figure 1A. C2C12 cell morphology staining after 3 days of culture in horse serum (Figure S1A). To investigate the potential interaction between ferroptosis and cuproptosis in the LCaRF model, we first confirmed that sorafenib (Sora) and erastin (Era), two representative ferroptosis inducers (FINs), inhibited C2C12 cell viability in a dose-dependent manner (Figure 1B). Notably, Sora and Era markedly sensitized C2C12 cells to Elesclomol–CuCl_2_ (Els–Cu)-induced cell death (Figure 1C). To exclude a compound-specific effect of Els, we further used diethyldithiocarbamate (DDC) as an alternative copper ionophore. Consistently, Sora and Era also potentiated DDC–Cu-induced cell death, which was similarly blocked by TTM (Figure 1E). In addition, Sora and Era increased Els- or DDC-induced intracellular lipid peroxidation in C2C12 cells (Figure 1G).

We next examined whether the FINs-mediated sensitization was associated with enhanced cuproptosis. Cuproptosis is characterized by the aggregation of lipoylated mitochondrial proteins, including dihydrolipoamide S-acetyltransferase (DLAT) [34]. Immunofluorescence analysis showed that Sora or Era combined with Els–Cu induced more pronounced DLAT aggregation than Els–Cu alone, and this effect was attenuated by TTM. Quantification of the DLAT aggregation index further supported these changes (Figure 1H and Figure S8A). In the DDC–Cu system, FIN-associated changes in DLAT aggregation were also assessed and attenuated by copper chelation (Figure 1H and Figure S8B). To further explore the involvement of copper-dependent mitochondrial dysfunction, we assessed cuproptosis-related gene expression following treatment with FINs alone or in combination with Els–Cu/DDC–Cu. qRT-PCR showed that Els–Cu combined with FINs increased several cuproptosis-sensitivity genes (*Fdx1*, *Lias*, *Lipt1*, *Dlat*, *Pdha1*, and *Pdhb*) and decreased genes related to copper export, metal buffering, or mitochondrial respiratory function (*Atp7a*, *Atp7b*, *Mtf1*, *Aco2*, *Atox1*, *Cutc*, and *Cox5b*); these alterations were reversed by TTM (Figure 1I). Similar results were observed using DDC–Cu, where FIN treatment increased the expression of cuproptosis marker genes, including *Dlat*, *Fdx1*, and *Lias*, in a concentration-dependent manner, and this effect was reversed by TEPA (Figure 1J). Collectively, these findings indicate that FINs potentiate copper-induced cell death in the LCaRF model through an intracellular copper-dependent mechanism.

### 3.2. Identification of Key Genes Mediating FIN-Enhanced Cuproptosis in LCaRF Cellular Models

**Figure 2:**
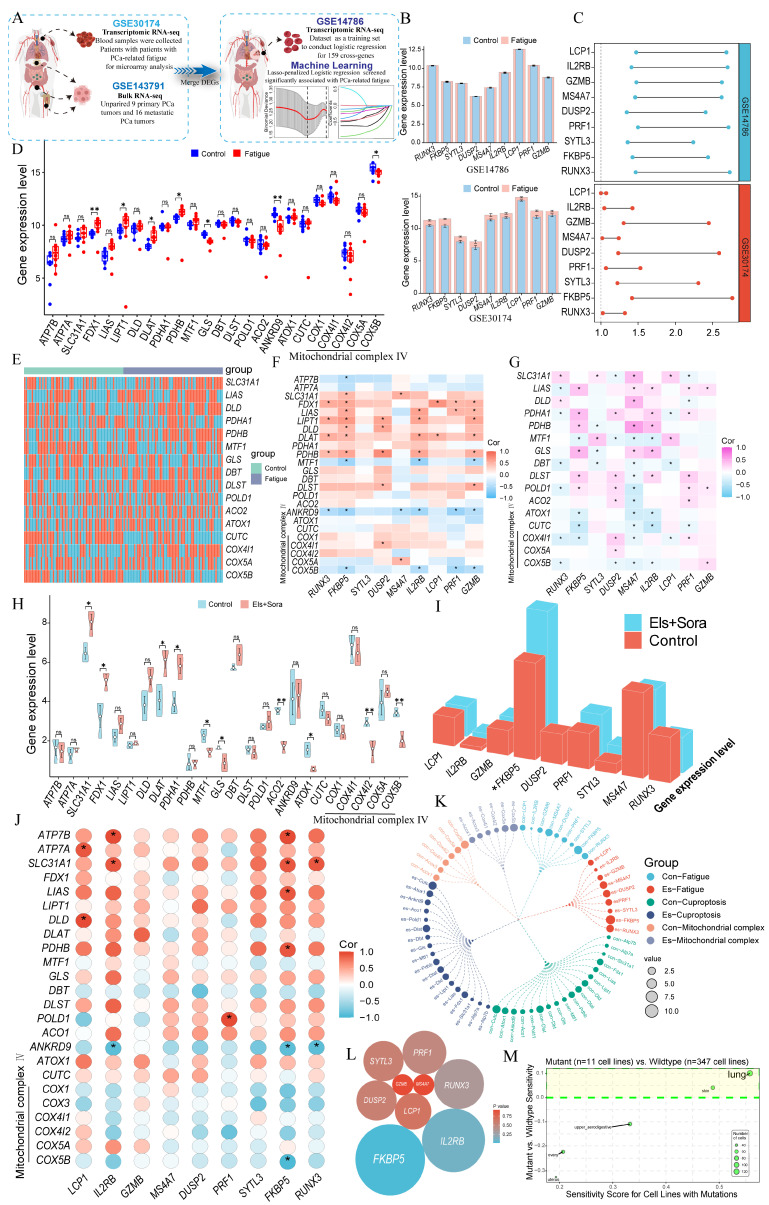
Identification of candidate genes associated with FIN-enhanced cuproptosis. (**A**). Flowchart depicting the screening process for candidate diagnostic marker genes associated with cancer-related fatigue (CRF). (**B**). Embedded bar charts showing dataset-specific expression levels of candidate CRF-associated diagnostic marker genes in GSE14786 and GSE30174. (**C**). Univariate logistic regression for candidate CRF diagnostic marker genes in the public dataset (GSE14786 or GSE30174). (**D**). The mRNA expression levels of cuproptosis-related genes in prostate cancer (PCa)-related fatigue utilizing the public dataset (GSE30174). (**E**). Heatmap depicting the expression distribution of cuproptosis-related genes across different samples in GSE14786 (red denotes high expression, while blue denotes low expression). (**F**,**G**). Dataset-specific correlation analyses between candidate CRF-associated diagnostic markers and cuproptosis-related genes in GSE30174 or GSE14786. (**H**). The mRNA expression levels of cuproptosis-related genes in LCaRF: Comparison between the 5 nM Els + 2.5 μM Sora treatment group and the untreated control group. (**I**). The mRNA expression levels of candidate CRF diagnostic marker genes in sequencing data from the LCaRF model. (**J**). Circular diagram of the correlation between candidate CRF diagnostic biomarkers and cuproptosis-related genes in LCaRF model. (**K**). The circular hierarchical graph depicts the weight distribution of genes across different groups within the RNA sequencing data associated with LCaRF. (**L**). Bubble chart showing the association between candidate CRF diagnostic marker genes and the average expression levels of mitochondrial respiratory chain complex (MRCC) IV-related genes derived from RNA-seq data on LCaRF. (**M**). DepMap analysis was used to identify the cancer type with the strongest *FKBP5*–*COX5B* association. Data are presented as mean ± SD. * *p* < 0.05, ** *p* < 0.01; ns, not significant.

To identify candidate genes associated with cancer-related fatigue (CRF), bulk RNA-seq data from three GEO datasets, GSE30174, GSE143791, and GSE14786, were analyzed [35,36,37] (Figure 2A). Based on our previous strategy [26], differentially expressed genes from GSE30174 were intersected with immune-related genes from GSE143791, and the resulting genes were subjected to LASSO regression using GSE14786 as the training dataset. To broaden the screening scope, genes excluded by LASSO regression were also retained for subsequent analysis. Finally, nine candidate CRF diagnostic marker genes were selected and evaluated in GSE14786 and GSE30174 (Figure 2B). Univariate logistic regression showed that these candidate diagnostic genes were positively associated with prostate cancer-related fatigue in GSE30174 and breast cancer-related fatigue in GSE14786 (Figure 2C).

Given the heterogeneity between GSE30174 and GSE14786, the two datasets were analyzed separately and used to evaluate fatigue-associated molecular patterns in prostate cancer- and breast cancer-related fatigue contexts, respectively. This design allowed us to assess whether candidate CRF-associated genes showed cross-context relevance rather than reflecting a single cancer type. We next analyzed cuproptosis-related gene expression in public datasets. In GSE30174, several cuproptosis-associated genes, including *FDX1*, *LIPT1*, *DLAT*, *PDHB*, *GLS*, *ANKRD9*, and *COX5B*, were significantly altered (Figure 2D). In GSE14786, high expression of *LIAS*, *DLD*, *PDHB*, *COX4I1*, and *DLST*, together with low expression of *MTF1*, *GLS*, *CUTC*, and *COX5B*, was predominantly enriched in the breast cancer-related fatigue group (Figure 2E). Correlation analysis further revealed regulatory associations among cuproptosis-related genes in GSE30174 (Figure S1B). In both GSE30174 and GSE14786, several candidate CRF diagnostic genes, including *RUNX3*, *FKBP5*, *DUSP2*, *IL2RB*, *GZMB*, *RUNX2*, *MS4A7*, and *PRF1*, were significantly correlated with multiple cuproptosis-related genes (Figure 2F,G), suggesting a potential link between candidate CRF diagnostic markers and cuproptosis.

To validate these associations in the LCaRF model, RNA sequencing was performed before and after Els–Cu + Sora treatment (Figure S1C,D). GO analysis showed that differentially expressed genes were mainly enriched in mitochondrial membrane function and cellular response to copper ions (Figure S1E–G). Several cuproptosis-related genes, including *SLC31A1*, *FDX1*, *DLAT*, *PDHA1*, *MTF1*, *GLS*, *ACO2*, *ATOX1*, *COX4I2*, and *COX5B*, were significantly altered (Figure 2H). Among the candidate CRF diagnostic genes, *Fkbp5* showed marked differential expression (Figure 2I). Correlation analysis demonstrated positive associations between candidate CRF markers, including *FKBP5*, *LCP1*, and *IL2RB*, and cuproptosis-related genes such as *ATP7A*, *ATP7B*, *SLC31A1*, *DLD*, *LIAS*, and *PDHB*, whereas *FKBP5* was negatively correlated with *COX5B* and *ANKRD9* (Figure 2J). Gene-weight analysis further identified *FKBP5*, *CUTC*, and *COX5B* as the most weighted fatigue-related, cuproptosis-related, and MRCC IV-related genes, respectively (Figure 2K). *FKBP5* showed the strongest negative correlation with the mean expression of MRCC IV-related genes (Figure 2L), which was validated in GSE30174 and GSE14786 (Figure S1I,J). DepMap analysis further showed that *FKBP5* was significantly associated with *COX5B* in lung cancer, whereas other candidate fatigue diagnostic genes showed no significant relationship with *COX5B* (Figure 2M and Figure S1H). In TCGA lung cancer cohorts, high *FKBP5* expression was associated with poorer overall survival (Figure S1K). These results suggest that FKBP5 is a candidate cuproptosis-associated CRF-related gene negatively linked to MRCC IV function and *COX5B*.

To determine whether FKBP5 participates in CIN + FIN-induced cell death, the expression of previously identified CRF diagnostic markers was examined by qRT-PCR. Els–Cu, DDC–Cu, or FINs alone increased *Fkbp5* expression, whereas combined Els–Cu/DDC–Cu + FIN treatment induced a more pronounced upregulation (Figure S3A), which was confirmed at the protein level by Western blotting (Figure S3B). This *FKBP5* induction was reversed by TTM or TEPA (Figure S2A–D). Because FINs such as sorafenib and erastin impair mitochondrial function by inhibiting system Xc− and inducing oxidative stress-mediated ferroptosis [38], we hypothesized that FINs would exacerbate oxidative stress in the LCaRF cell model. As shown in Figure S2E, treatment with Els–Cu (or DDC–Cu) combined with FINs reduced SOD activity and increased MDA levels, and these effects were reversed by TTM (or TEPA). Further analysis revealed that the activity of MRCC IV was also significantly inhibited by the combination treatment and restored by TTM (or TEPA) (Figure S2F). When copper is transported into cells by CINs, *FDX1* promotes cuproptosis by reducing Cu2+ to Cu+ in mitochondria, enabling Cu+ to bind lipoylated TCA-cycle proteins, induce their aggregation, and destabilize Fe-S cluster proteins [39]. Although Fe-S-cluster protein loss is a canonical downstream feature of cuproptosis, FDX1 and LIAS are upstream determinants of cuproptosis sensitivity; their depletion generally reduces protein lipoylation and confers resistance rather than serving as the initiating injury event [40]. Consistent with reports that FINs can stabilize FDX1 and enhance CIN-induced cuproptosis [23], IF analysis revealed that FDX1 expression was significantly upregulated following Els–Cu (or DDC–Cu) combined with FINs, and this effect was reversed by TTM (or TEPA) (Figure S2G). Quantification of relative FDX1 fluorescence intensity further supported these changes in both Els–Cu- and DDC–Cu-based systems (Figure S8M,N). TMRM and ATP assays showed that Els–Cu/DDC–Cu + FINs reduced mitochondrial membrane potential and ATP synthesis in C2C12 cells, effects reversed by TTM/TEPA (Figures S2H and S3D). Moreover, ROS levels increased with Els–Cu/DDC–Cu + FINs and were reversed by TTM/TEPA (Figure S2H). Among MRCC IV-related genes, *COX5B*—identified as being significantly negatively correlated with *FKBP5*—plays a crucial role in the regulation of mitochondrial function and in oxidative damage repair [41,42]. Western blotting results confirmed that treatment with Els–Cu + FINs reduced the expression of lipoylated DLAT, lipoylated DLST, and COX5B, while increasing FKBP5 expression (Figure S2I,J). The above results further indicate that Els–Cu/DDC–Cu + FINs-induced cell death involves ROS generation and mitochondrial damage, potentially mediated by *FKBP5*.

### 3.3. FINs Promote Cuproptosis by Depleting Intracellular GSH in LCaRF Cellular Models

**Figure 3:**
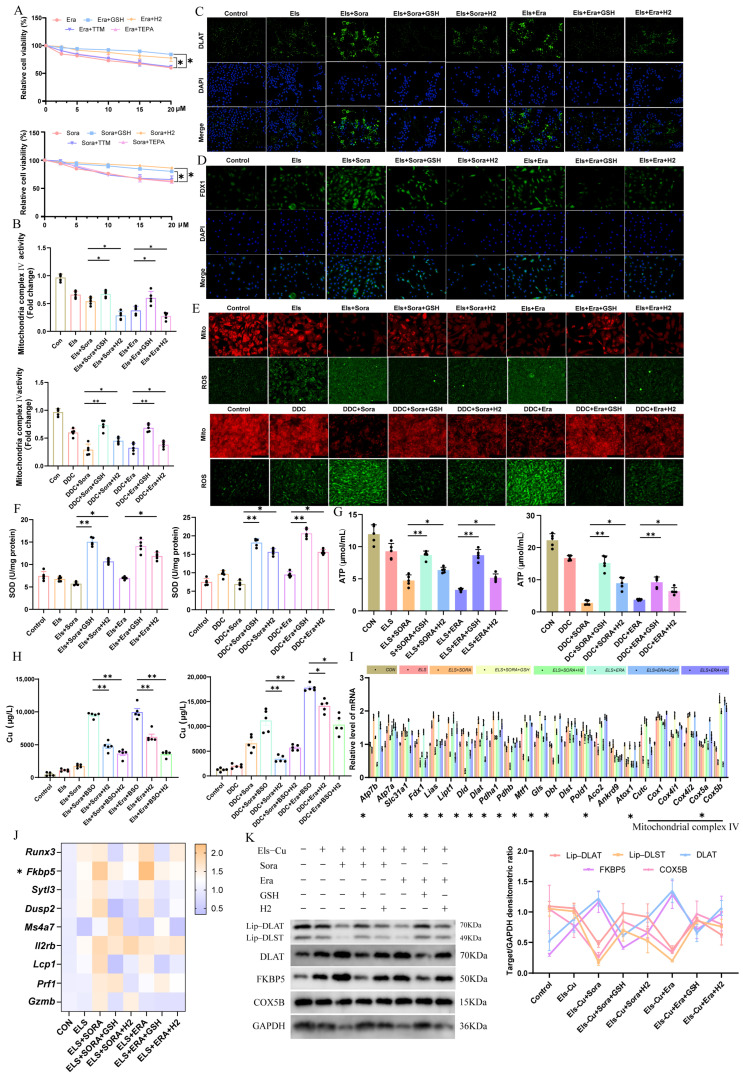
FINs promoted cuproptosis by depleting intracellular GSH. (**A**). Cell viability of C2C12 after Era or Sora treatment, together with DMSO, 5 μM TTM, 5 mM GSH, hydrogen, or 2.5 mM TEPA together was measured with a CCK-8 assay. (**B**). The MRCC IV activity in C2C12 cells after Els 24 h treatment, together with 2.5 μM Sora (or 2.5 μM Era), 2.5 μM Sora (or 2.5 μM Era) +5 μM TTM, 2.5 μM Sora (or 2.5 μM Era) + 5 mM GSH or 2.5 μM Sora (or 2.5 μM Era) + hydrogen together was measured. (**C**,**D**). C2C12 cells were treated with indicated drugs for 24 h, followed by IF analysis to assess DLAT aggregation or FDX1 expression (green, DLAT or FDX1; blue, DAPI). Scale bar: 100 μm. (**E**). Assessment of TMRM uptake for mitochondrial membrane potential and ROS levels in LCaRF models after indicated drug treatment. Scale bar: 250 μm. (**F**,**G**). SOD activity and ATP levels were detected in the LCaRF model after the indicated treatments. (**H**). Detection of copper levels in C2C12 cells was measured after 24 h treatment with 5 nM Els or 50 nM DDC, along with DMSO, 2.5 μM Sora (or 2.5 μM Era), 2.5 μM Sora (or 2.5 μM Era) + 1 mM L-buthionine sulfoximine (BSO) or 2.5 μM Sora (or 2.5 μM Era) + 1 mM BSO + hydrogen. (**I**,**J**). The mRNA expression levels of cuproptosis-related genes (**I**) or CRF diagnostic marker genes (**J**) in C2C12 cells after indicated drug treatment for 24 h in the LCaRF model. (**K**). The protein levels of lipoylated DLAT, lipoylated DLST, DLAT, FKBP5, and COX5B in LCaRF models after indicated drug treatment. Western blot quantification is presented as Target/GAPDH densitometric ratios. For panels (**A**–**K**), media were supplemented with 1 µM CuCl_2_. Data are presented as mean ± SD from three independent biological experiments unless otherwise indicated. Indicated comparisons are shown in the corresponding panels. * *p* < 0.05, ** *p* < 0.01.

This enhanced cytotoxicity was reversed by the copper chelators TTM and TEPA (Figure 1D), whereas TTM or TEPA did not attenuate cell death induced by Sora or Era alone (Figure 3A). Given that GSH can suppress cuproptosis by chelating copper [39] and hydrogen can selectively scavenge cytotoxic hydroxyl radicals [43], we next examined whether GSH depletion and oxidative stress contribute to FIN-mediated cuproptosis sensitization. GSH or hydrogen reversed FIN-induced cell death, suggesting their potential involvement in this process (Figure 3A). Sora or Era, either alone or combined with Els–Cu/DDC–Cu, significantly reduced intracellular GSH levels (Figure S3E,G). Supplementation with GSH or hydrogen reversed FIN-enhanced copper-induced cytotoxicity (Figure S3J,K), reduced DLAT aggregation, as confirmed by DLAT aggregation index quantification (Figure 3C, Figures S3L and S8C,D), and decreased FDX1 expression under Els–Cu + FIN treatment (Figure 3D). Quantification of relative FDX1 fluorescence intensity further supported the suppressive effects of GSH or H_2_ on FIN-enhanced FDX1 upregulation in both Els–Cu- and DDC–Cu-based systems (Figure S8O,P). Similar results were obtained using DDC–Cu (Figure S3M).

GSH or hydrogen also alleviated Els–Cu/DDC–Cu + FIN-induced mitochondrial dysfunction, as indicated by improved mitochondrial membrane potential, increased ATP levels, and restored MRCC IV activity (Figure 3B,E,G). Oxidative stress assays showed that GSH or hydrogen reduced ROS and MDA accumulation and restored SOD activity (Figure 3E,F and Figure S2H). In contrast, BSO-mediated GSH depletion further increased intracellular copper accumulation, whereas GSH or hydrogen decreased copper levels (Figure 3H). At the transcriptional level, GSH or hydrogen reversed Els–Cu + FIN-induced upregulation of cuproptosis-sensitivity genes, including *Fdx1*, *Lias*, *Lipt1*, *Dld*, *Dlat*, *Pdhb*, and *Pdha1*, and restored genes related to metal buffering or mitochondrial homeostasis, including *Mtf1, Gls, Aco2, Cutc,* and *Cox5b* (Figure 3I). Similar effects were observed under DDC–Cu + FIN treatment (Figure S3I). Moreover, GSH or hydrogen reversed *Fkbp5* upregulation induced by Els–Cu/DDC–Cu + FINs (Figure 3J and Figure S3C), and Western blotting confirmed restoration of lipoylated DLAT, lipoylated DLST, and COX5B, along with reduced FKBP5 expression (Figure 3K). These findings indicate that FINs promote cuproptosis in LCaRF cellular models, at least in part, by depleting intracellular GSH.

### 3.4. FKBP5 Contributes to FIN-Induced Sensitization to Cuproptosis in LCaRF Cellular Models

To clarify whether cuproptosis overlaps with other cell death modalities under Els–Cu/DDC–Cu + FIN treatment, we examined the effects of multiple cell death inhibitors. TTM partially restored cell viability even in the presence of Z-VAD-fmk, ferrostatin-1, or Nec-1s, either alone or in combination, supporting a predominant contribution of cuproptosis (Figure S4A). *Fkbp5* knockdown also restored cell viability under treatment with Z-VAD-fmk, ferrostatin-1, or Nec-1s, but not with TTM, suggesting that FKBP5 functions upstream of copper-dependent cell death (Figure S4B).

**Figure 4:**
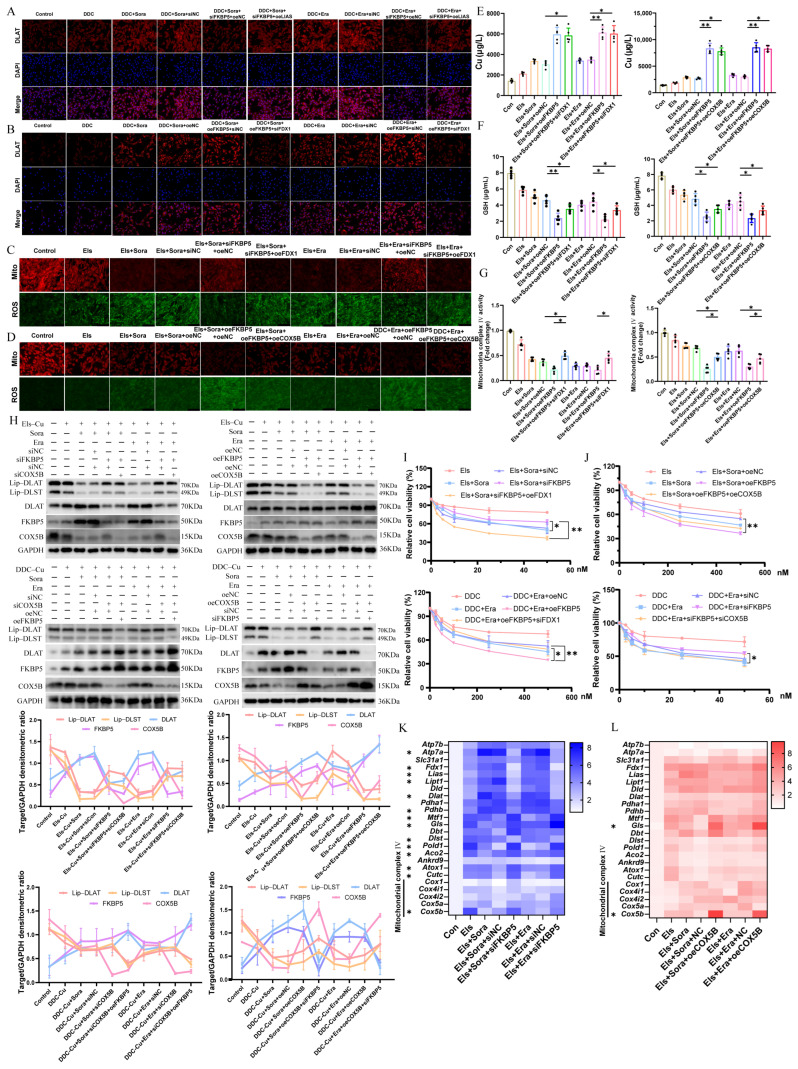
*FKBP5* contributes to FIN-enhanced cuproptosis. (**A**,**B**). Following the intervention of *Fkbp5*, *Lias*, or *Fdx1* expression, C2C12 cells were treated with indicated drugs for 24 h (DMSO, 50 nM DDC, 50 nM DDC + 2.5 µM Sora (or 2.5 μM Era)), and DLAT protein aggregation was analyzed by IF (red, DLAT; blue, DAPI). Scale bar: 100 μm. (**C**,**D**). Assessment of TMRM uptake for mitochondrial membrane potential and ROS levels in LCaRF models after *Fkbp5*, *Cox5b*, or *Fdx1* expression interference and drug treatment. Scale bar: 250 μm. (**E**–**G**). Detection of copper levels (**E**), glutathione (GSH) content (**F**) and the activity of MRCC IV (**G**) in LCaRF models after *Fkbp5*, *Cox5b*, or *Fdx1* expression interference and drug treatment. (**H**). The protein levels of lipoylated DLAT, lipoylated DLST, DLAT, FKBP5, and COX5B in C2C12 cells in LCaRF models after *Fkbp5* and *Cox5b* expression interference and drug treatment. Western blot quantification is presented as Target/GAPDH densitometric ratios. (**I**,**J**). Cell viability of C2C12 cells following *Fkbp5*, *Cox5b*, *or Fdx1* gene expression interference and pharmacological treatment in cellular models of LCaRF. (**K**,**L**). The mRNA expression of C2C12 cells after indicated drug treatment for 24 h under *Fkbp5* or *Cox5b* regulation. For panels (**A**–**L**), media were supplemented with 1 µM CuCl_2_. Data are presented as mean ± SD from three independent biological experiments unless otherwise indicated. Indicated comparisons are shown in the corresponding panels. * *p* < 0.05, ** *p* < 0.01.

Functional assays further demonstrated that *Fkbp5* overexpression enhanced DLAT aggregation, whereas *Fkbp5* knockdown reduced it. Quantification of the DLAT aggregation index confirmed these effects in both Els–Cu- and DDC–Cu-based systems (Figure 4A,B and Figure S8E–H). Rescue analyses further showed that *Lias* or *Fdx1* modulation altered the DLAT aggregation changes induced by *Fkbp5* knockdown or overexpression (Figures S4C,D and S8G,H). *Fkbp5* overexpression aggravated Els–Cu/DDC–Cu + FIN-induced mitochondrial membrane potential loss and ROS generation, whereas *FDX1* or *COX5B* intervention reversed these effects (Figure 4C,D and Figure S4E,F). GSH and MRCC IV assays yielded consistent results (Figure 4F,G and Figure S4G,I). Copper quantification showed that modulation of *FDX1* or *COX5B* did not reduce FKBP5-enhanced copper accumulation under Els–Cu/DDC–Cu + FIN treatment (Figure 4E and Figure S4H), suggesting that FKBP5 acts downstream of copper accumulation to aggravate mitochondrial injury.

Cell viability assays showed that *Fkbp5* overexpression exacerbated DDC–Cu + Era-induced cytotoxicity, whereas *FDX1* knockdown reversed this effect (Figure 4I). Similarly, *COX5B* overexpression alleviated FKBP5-enhanced Els–Cu + Sora-induced injury (Figure 4J). Western blotting showed that *Fkbp5* overexpression reduced COX5B and the detectable soluble/monomeric lipoylated DLAT and lipoylated DLST signals, whereas *Fkbp5* knockdown increased these signals; this pattern is consistent with copper-induced aggregation or redistribution of lipoylated proteins rather than simple loss of the lipoylated substrate pool [13,39]. In contrast, COX5B modulation did not affect FKBP5, lipoylated DLAT, or lipoylated DLST levels (Figure 4H). Immunofluorescence analysis further demonstrated that genetic knockdown of LIAS or FDX1 did not markedly reduce FKBP5 expression following combined treatment with Els–Cu/DDC–Cu + FINs (Figure S5A,B), which was further supported by quantification of relative FKBP5 fluorescence intensity (Figure S8S,T). At the mRNA level, *Fkbp5* knockdown reduced cuproptosis-sensitivity genes, including *Fdx1*, *Lias*, *Lipt1*, *Dlat, Pdhb*, and *Dlst*, while increasing genes involved in copper transport, mitochondrial metabolism, or stress buffering, including *Atp7a*, *Pold1, Aco2*, *Atox1*, *Cutc*, *Mtf1*, *Gls*, and *Cox5b* (Figure 4K). *COX5B* overexpression mainly increased *GLS* expression under the same conditions (Figure 4L). Together, these data indicate that FKBP5 is a critical mediator of FIN-enhanced CIN-induced cuproptosis.

### 3.5. FKBP5-Associated Nrf2/HO-1 Suppression Contributes to FIN-Induced Cuproptosis Sensitization

**Figure 5:**
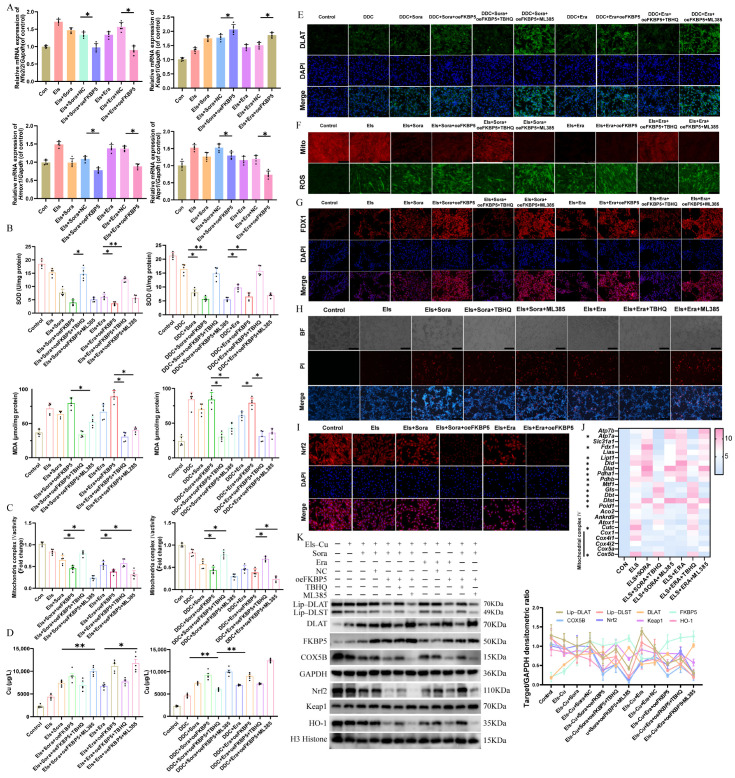
FKBP5-associated impairment of Nrf2/HO-1 signaling contributes to FIN-enhanced cuproptosis sensitization. (**A**). The mRNA expression levels of genes related to the Nrf2/HO-1 pathway in C2C12 cells after 24 h treatment with the specified drug in the LCaRF model. (**B**–**D**). Detection of the levels of SOD, MDA, MRCC IV activity and copper levels in C2C12 cells treated with indicated drugs (DMSO, 5 nM Els/50 nM DDC, 5 nM Els/50 nM DDC + 2.5 µM Sora, 5 nM Els/50 nM DDC + 2.5 µM Sora + 5 μM tertiary butylhydroquinone (TBHQ), 5 nM Els/50 nM DDC + 2.5 µM Sora + 1 μM ML385, 5 nM Els/50 nM DDC + 2.5 µM Era, 5 nM Els/50 nM DDC + 2.5 µM Era  +  5 μM TBHQ, 5 nM Els/50 nM DDC + 2.5 µM Era + 1 μM ML385). (**E**,**G**). The IF images of DLAT (**E**) or FDX1 (**G**) in C2C12 cells treated with indicated drugs in the LCaRF model (red, FDX1; green, DLAT; blue, DAPI). Scale bar: 100 μm. (**F**). Assessment of TMRM uptake for mitochondrial membrane potential and ROS levels in LCaRF models. Scale bar: 250 μm. (**H**). The Hoechst 33342/PI double-stained images of C2C12 cells treated with indicated drugs in the LCaRF model (red, PI; blue, Hoechst 33342). Scale bar: 100 μm. (**I**). The IF images of Nrf2 in C2C12 cells treated with drugs (DMSO, 5 nM Els, 5 nM Els + 2.5 µM Sora, 5 nM Els + 2.5 µM Era) in the LCaRF model (red, Nrf2; blue, DAPI). Scale bar: 100 μm. (**J**). The mRNA expression levels of cuproptosis-related genes in C2C12 cells after indicated drug 24 h treatment in the LCaRF model. (**K**). The protein levels of lipoylated DLAT, lipoylated DLST, DLAT, FKBP5, and COX5B in LCaRF models after indicated drug treatment. Western blot quantification is presented as Target/GAPDH densitometric ratios. For panels (**A**–**K**), media were supplemented with 1 µM CuCl_2_. Data are presented as mean ± SD from three independent biological experiments unless otherwise indicated. Indicated comparisons are shown in the corresponding panels. * *p* < 0.05, ** *p* < 0.01.

To investigate the mechanism underlying FKBP5-mediated sensitization to cuproptosis, KEGG enrichment analysis was performed using transcriptomic DEGs. The results showed prominent enrichment of the Nrf2/HO-1 signaling pathway (Figure S5C). Under Els–Cu + FIN treatment, *Fkbp5* overexpression markedly downregulated Nrf2/HO-1 pathway-related genes, including *Nfe2l2, Hmox1,* and *Nqo1*, while upregulating *Keap1* (Figure 5A). Functionally, TBHQ, an Nrf2 activator that induces downstream antioxidant programs including *HO-1*, reversed *Fkbp5* overexpression-induced suppression of SOD activity and elevation of MDA levels, whereas ML385, an Nrf2 inhibitor, further aggravated these effects (Figure 5B). Similar protective effects of TBHQ were observed for mitochondrial membrane potential, MRCC IV activity, and intracellular copper accumulation (Figure 5C,D,F and Figure S6D).

Immunofluorescence showed that TBHQ reversed FKBP5-enhanced DLAT aggregation induced by Els–Cu/DDC–Cu + FINs, which was further confirmed by DLAT aggregation index quantification (Figure 5E, Figures S6A and S8I,J). *Fkbp5* overexpression further upregulated FDX1 expression under combined treatment, whereas TBHQ attenuated this effect (Figure 5G and Figure S6B). Quantification of relative FDX1 fluorescence intensity further supported these changes in both Els–Cu- and DDC–Cu-based systems (Figure S8Q,R). Hoechst 33342/PI staining showed increased PI-positive cells after Els–Cu/DDC–Cu + FIN treatment, which was reduced by TBHQ (Figure 5H and Figure S6C). Nrf2 immunofluorescence revealed that Els–Cu + FINs promoted Nrf2 nuclear translocation, whereas Fkbp5 overexpression reduced nuclear Nrf2 accumulation; this pattern was confirmed by quantification of the Nrf2 nuclear/cytoplasmic fluorescence ratio (Figure 5I and Figure S8K). Furthermore, TBHQ reduced the expression of cuproptosis-sensitivity genes, including *Fdx1*, *Lipt1*, *Dld*, *Dlat*, *Pdha1*, *Pdhb*, and *Dlst*, while increasing genes linked to metal buffering, mitochondrial metabolism, or stress resistance, such as *Mtf1*, *Gls*, *Dbt*, *Pold1*, *Cutc*, and *Cox5b* (Figure 5J). Comparable results were obtained under DDC–Cu + FIN treatment (Figure S5D). Western blotting confirmed that *Fkbp5* overexpression suppressed Nrf2 and HO-1 protein expression, whereas TBHQ restored COX5B, lipoylated DLAT, and lipoylated DLST levels (Figure 5K). Co-IP assays showed no clear FKBP5–Nrf2 co-precipitation despite marked FKBP5 enrichment after *Fkbp5* overexpression (Figure S7A), suggesting that FKBP5-associated Nrf2/HO-1 suppression may occur indirectly. To further assess the role of Nrf2-associated antioxidant signaling, we combined *Fkbp5* knockdown with ML385 treatment under Els–Cu + Sora exposure. *Fkbp5* knockdown improved cell viability, reduced DLAT aggregation, and increased the expression of the Nrf2 target genes *Hmox1* and *Nqo1*, whereas ML385 attenuated these effects (Figure S7B–F). These results suggest that *Fkbp5* knockdown alleviates FIN–CIN–Cu-induced cuproptosis-related injury at least partly through Nrf2-associated antioxidant signaling.

### 3.6. Combined FIN and CIN Treatment Exacerbated Fatigue-like Behaviors in LCa-Bearing Mice

**Figure 6:**
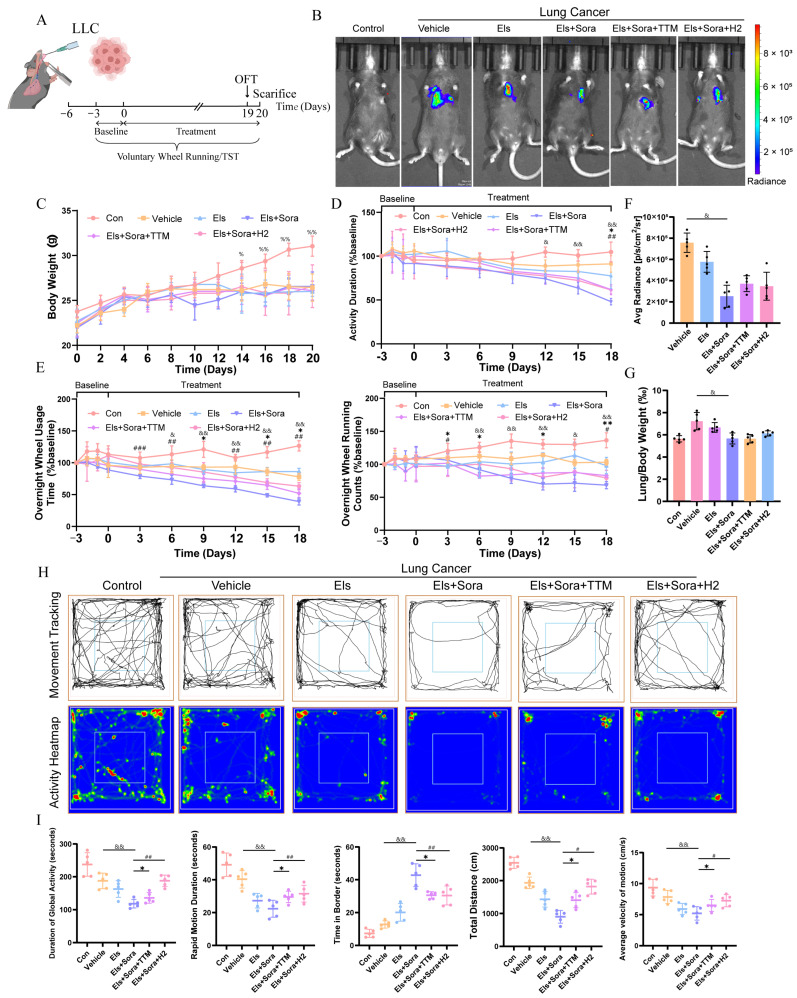
FINs exacerbate fatigue-like behaviors in mice with orthotopic lung cancer. (**A**). Experimental design and timeline. (**B**,**F**). Representative bioluminescence imaging of orthotopic lung cancer and quantification of average radiance in mice. (**C**–**E**). Body weight (**C**), tail suspension activity duration (**D**), and overnight voluntary wheel running duration and revolutions during a 12 h dark-cycle recording period (**E**) in LCa-bearing mice. (**G**). Lung wet weight-to-body weight ratio in LCa-bearing mice. (**H**). Representative heatmaps and trajectories from the open field test (OFT). The outer square indicates the boundary of the open-field arena, and the inner blue square indicates the center zone used for behavioral analysis. Black lines represent movement trajectories, and heatmap colors indicate activity density. (**I**). Quantification of global activity duration, rapid movement duration, border-zone duration, total distance, and average velocity in the OFT. Data are presented as mean ± SD; *n* = 5 mice per group. Longitudinal data in panels (**C**–**E**) were analyzed using mixed-effects analysis with treatment group and time as factors, followed by Šídák-adjusted post hoc comparisons at individual time points. Endpoint measurements in panels (**F**,**G**,**I**) were analyzed using one-way ANOVA followed by Tukey’s multiple-comparison test. For panels (**C**–**G**,**I**), % *p* < 0.05 and %% *p* < 0.01 indicate Els–Cu + Sora versus Con; & *p* < 0.05 and && *p* < 0.01 indicate Els–Cu + Sora versus Vehicle; * *p* < 0.05 and ** *p* < 0.01 indicate Els–Cu + Sora + TTM versus Els–Cu + Sora; # *p* < 0.05, ## *p* < 0.01 and ### *p* < 0.001 indicate Els–Cu + Sora + H_2_ versus Els–Cu + Sora.

An orthotopic lung cancer model was used to evaluate the in vivo effects of Els–Cu + FINs on fatigue-like behaviors and skeletal muscle injury. As shown in Figure 6A, fatigue-like behaviors were assessed using voluntary wheel running, the tail suspension test, and the open field test. Bioluminescence imaging was performed 20 days after intervention (Figure 6B). Tumor burden was reduced in intervention groups compared with vehicle controls, while TTM or hydrogen did not significantly alter tumor growth compared with the Els–Cu + Sora group (Figure 6F,G). Body weight decreased from day 12 onward in LCa-bearing mice, likely due to progressive tumor burden (Figure 6C).

Baseline voluntary wheel running and tail suspension performance did not differ among groups. Longitudinal analysis using a mixed-effects model showed treatment-dependent changes in body weight, voluntary wheel running duration, wheel revolutions, and tail suspension performance over time (Figure 6C–E). Els–Cu + Sora progressively reduced voluntary wheel running and tail suspension performance compared with vehicle-treated LCa-bearing mice, whereas TTM or H_2_ partially improved these behavioral trajectories. Post hoc comparisons at individual time points further supported the protective effects of TTM and H_2_ compared with Els–Cu + Sora alone (Figure 6D,E). OFT analysis further showed that TTM or H_2_ improved Els–Cu + Sora-induced reductions in locomotor activity, including global activity duration, rapid movement duration, total distance, and average velocity, while reducing border-zone duration (Figure 6H,I). These results indicate that CINs combined with FINs exacerbate fatigue-like behaviors in LCa-bearing mice, whereas copper chelation or hydrogen supplementation partially mitigates these effects.

### 3.7. FINs Promoted Cuproptosis in LCa-Bearing Mice

**Figure 7:**
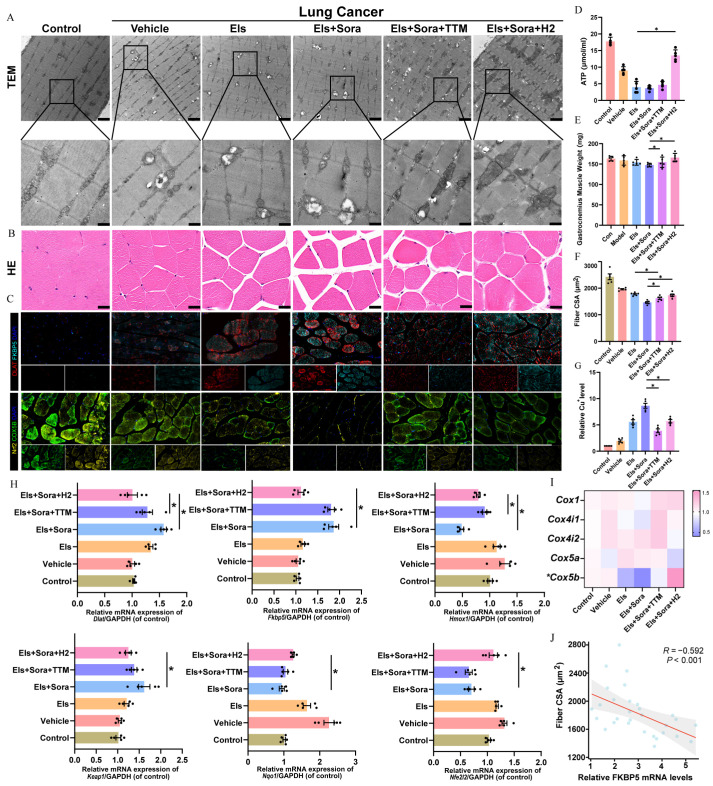
Combined FIN and CIN–Cu treatment is associated with cuproptosis-related skeletal muscle injury in mice with orthotopic lung cancer. (**A**). Transmission electron microscopy observation of gastrocnemius muscle mitochondria in LCa-bearing mice. Scale bar: 2 μm, 1 μm. (**B**). Representative H&E-stained images of gastrocnemius muscle from LCa-bearing mice. Scale bar: 20 μm. (**C**). Representative images of IF staining of the gastrocnemius muscle from LCa-bearing mice are shown (red, DLAT; cyan, FKBP5; green, COX5B; yellow, Nrf2; blue, DAPI). Scale bar: 20 μm. (**D**–**G**). ATP content, muscle weight, muscle fiber cross-sectional area (CSA), and copper ion levels in the gastrocnemius muscle of LCa-bearing mice. (**H**). *Dlat*, *Fkbp5*, *Hmox1*, *Keap1*, *Nqo1*, and *Nfe2l2* gene expression changes were assessed by qRT-PCR. (**I**). Expression of MRCC IV -related genes was assessed by RT-qPCR. (**J**). Scatter plot showing the association between *Fkbp5* expression in lung tumor tissue and muscle fiber CSA. Red line, fitted regression line; grey area, 95% confidence interval. Data are presented as mean ± SD; *n* = 5 mice per group unless otherwise indicated. Indicated comparisons are shown in the corresponding panels. * *p* < 0.05.

Based on the in vitro findings, we further examined the in vivo effects of combined Els–Cu and Sora treatment. Transmission electron microscopy of gastrocnemius muscle showed that Els–Cu + Sora induced disorganized myofilaments, loosened myofibrils, reduced and irregular mitochondria, disrupted or absent cristae, and intramitochondrial vacuolization compared with controls. These abnormalities were alleviated by TTM or hydrogen supplementation (Figure 7A). Consistently, TTM or hydrogen, particularly hydrogen, increased ATP production compared with Els–Cu + Sora alone (Figure 7D). Both interventions also increased gastrocnemius muscle mass (Figure 7E).

H&E staining revealed that Els–Cu + Sora caused gastrocnemius muscle fiber atrophy, as evidenced by reduced cross-sectional area (CSA), whereas TTM or hydrogen mitigated this atrophic change (Figure 7B,F). Copper content analysis showed that TTM significantly reduced Els–Cu + Sora-induced copper accumulation in gastrocnemius muscle (Figure 7G). At the protein level, Els–Cu + Sora increased DLAT and FKBP5 while reducing Nrf2 and COX5B expression; these changes were attenuated by TTM or H_2_ (Figure 7C). Quantitative fluorescence analysis further showed that Els–Cu + Sora increased the DLAT aggregation index and FKBP5 fluorescence intensity, while decreasing COX5B and Nrf2 fluorescence intensities in gastrocnemius muscle. TTM or H_2_ partially attenuated these changes (Figure S8L,U–W). mRNA analysis further showed that TTM or hydrogen attenuated the inhibitory effects of Els–Cu + FINs on *Cox5b* and Nrf2 pathway genes, including *Nfe2l2*, *Hmox1*, and *Nqo1* (Figure 7H,I). Correlation analysis revealed that *Fkbp5* expression in orthotopic lung cancer tissues was negatively correlated with gastrocnemius CSA (R = −0.592) (Figure 7J). Collectively, these results suggest that combined FIN and CIN–Cu treatment is associated with enhanced cuproptosis-related changes and skeletal muscle injury in LCa-bearing mice.

## 4. Discussion

As cancer incidence increases, CRF has become an important public health issue requiring improved diagnosis and mechanistic understanding. CRF is defined as persistent, distressing, and disproportionate physical, emotional, or cognitive fatigue related to cancer or its treatment that interferes with daily functioning [1]. Previous studies have reported that more than 90% of patients receiving nonsurgical treatment and up to 82% of LCa survivors experience CRF [44]. With the discovery of ferroptosis and cuproptosis and the development of targeted agents that modulate ion homeostasis, the mechanisms underlying CRF have become increasingly complex [13,45]. In this study, we found that FINs (Sora and Era) enhanced CIN–Cu-induced cuproptosis and skeletal muscle injury in LCaRF models, accompanied by intracellular GSH depletion, *Fkbp5* upregulation, and impaired Nrf2/HO-1 antioxidant signaling (Figure S9). These findings do not support a strictly linear pathway. Instead, FINs may first deplete GSH and increase oxidative stress, thereby inducing FKBP5 as part of a cellular stress response. Elevated FKBP5 may then act as a stress-amplifying mediator that further weakens Nrf2/HO-1-dependent antioxidant defense, promoting ROS accumulation, mitochondrial dysfunction, and cuproptosis-related injury. Consistently, our Co-IP assay did not provide evidence for a stable direct FKBP5–Nrf2 interaction (Figure S7), suggesting that FKBP5-associated Nrf2/HO-1 suppression may occur indirectly, potentially through GR-related signaling, Keap1/Nrf2 homeostasis, or other stress-responsive pathways [46,47]. Additional rescue/epistasis analysis further supported this interpretation. *Fkbp5* knockdown reduced DLAT aggregation and improved cell viability under Els–Cu + Sora treatment, whereas ML385 partially attenuated these protective effects. In parallel, *Fkbp5* knockdown increased the expression of the Nrf2 target genes *Hmox1* and *Nqo1*, while ML385 suppressed their expression. Together with the negative Co-IP result, these findings support an indirect FKBP5-associated impairment of Nrf2/HO-1 signaling rather than a stable direct FKBP5–Nrf2 interaction.

CRF involves both central and peripheral mechanisms [48]. Central fatigue arises from impaired CNS signaling or reduced excitability, whereas peripheral fatigue reflects the inability of skeletal muscle to sustain contraction due to neuromuscular dysfunction, mitochondrial impairment, or accumulation of metabolic byproducts [5]. Mitochondrial structural damage, oxidative stress, and ATP depletion are key contributors to CRF [49]. Because skeletal muscle undergoes myogenic proliferation and differentiation after cuproptosis, myocytes provide a suitable model for evaluating the impact of CIN–Cu plus FINs on muscle homeostasis [50]. Based on previous work, we established an in vitro LCaRF model using C2C12 cells exposed to LLC- or M109-derived paracrine factors [20].

Copper and iron are essential cofactors involved in antioxidant defense, mitochondrial respiration, angiogenesis, and tumor progression [51]. Targeting metal metabolism has been translated into cancer therapy, as shown by sorafenib in LCa [52] and Els-mediated copper-dependent cell death in esophageal cancer [40]. Crosstalk among regulated cell death pathways, including necroptosis, pyroptosis, ferroptosis, and cuproptosis, is increasingly recognized [51], and combination strategies targeting distinct cell death programs are emerging as promising anticancer approaches. Wang et al. reported that FINs enhanced CIN-induced cuproptosis in hepatocellular carcinoma by inhibiting FDX1 degradation [23]. Consistently, we found that FINs (Sora or Era) aggravated CIN-induced mitochondrial dysfunction, oxidative stress, and cell death, thereby disrupting muscle homeostasis and peripheral fatigue-like phenotypes.

GSH acts as both a copper chelator controlling the labile copper pool [39] and a GPX4 substrate required for lipid peroxide reduction. Sora and Era induce ferroptosis mainly by inhibiting SLC7A11, the catalytic subunit of system Xc− responsible for cystine uptake and GSH synthesis [38]. In the LCaRF model, FINs alone or combined with CINs markedly reduced intracellular GSH levels. Because GSH is rapidly depleted and difficult to replenish, we explored alternative strategies to attenuate FIN- and CIN-induced cuproptosis. Hydrogen, a novel antioxidant, has been widely used to alleviate oxidative stress in plants and medicine [43], and hydrogen-rich water with nanobubbles was reported to reduce copper toxicity in aquatic organisms [53]. Although less effective than GSH, hydrogen supplementation reduced FIN- and CIN-induced copper toxicity and improved fatigue-like behaviors in tumor-bearing mice.

*Fkbp5* upregulation has been linked to type 2b skeletal muscle atrophy in chronic stress models [54] and to ischemic cardiomyopathy [55], and *FKBP5* may cooperate with Sora to inhibit liver cancer metastasis [56]. However, its role in ion homeostasis and CRF remains unclear. We showed that FKBP5 was markedly upregulated after FIN and CIN treatment and aggravated CIN-induced copper toxicity. Cytochrome c oxidase (COX), the terminal enzyme of MRCC IV, contains CuA and CuB centers essential for electron transfer and proton-gradient formation [57], and disrupted copper homeostasis impairs COX function, particularly in muscle cells [58]. Correlation and gene expression analyses revealed a significant negative association between *COX5B* and *FKBP5*, suggesting that FIN- and CIN-induced muscle dysfunction may partly involve impaired COX activity. The Nrf2/HO-1 pathway is a classical antioxidant defense mechanism involved in multiple regulated cell death pathways [59,60,61], and recent evidence links Nrf2 activation and GSH metabolism to resistance against copper-induced proteotoxic stress [62]. Here, TBHQ attenuated FKBP5-overexpression-induced cuproptosis under combined FIN and CIN exposure.

Because this study was based on lung cancer models, hydrogen-based supportive intervention for FIN–CIN-associated fatigue-like phenotypes should first be evaluated in this context. The in vivo findings should be interpreted as evidence of peripheral muscle injury and fatigue-like behaviors in LCaRF models, rather than definitive clinical CRF causation. Further studies are needed to determine whether these mechanisms and interventions apply to other cancers, and clinical validation is required before translation. The absence of sorafenib-alone, non-tumor-bearing drug-treated, and full factorial FIN/CIN control groups further limits the ability to distinguish tumor effects, drug toxicity, CIN effects, FIN effects, and their interaction. Although GSE30174 and GSE14786 represent different cancer-related fatigue contexts, they were analyzed independently and used as complementary validation cohorts rather than merged for direct comparison. Therefore, the shared findings were interpreted as candidate CRF-associated molecular patterns and were subsequently investigated in the LCaRF experimental models.

## 5. Conclusions

Overall, our findings suggest crosstalk between ferroptosis and cuproptosis in LCaRF experimental models. FINs enhanced CIN–Cu-associated skeletal muscle injury primarily through GSH depletion and oxidative stress, while stress-induced *Fkbp5* upregulation further impaired Nrf2/HO-1-associated antioxidant defense and amplified cuproptosis-related mitochondrial injury. The combination of FINs and CIN–Cu was associated with peripheral fatigue-like phenotypes, whereas hydrogen partially mitigated these effects. Further studies with additional in vivo control groups, direct mechanistic validation, and clinical cohorts are required before definitive CRF causation or therapeutic translation can be inferred.

## Data Availability

The publicly available datasets analyzed in this study are openly available in the Gene Expression Omnibus database at https://www.ncbi.nlm.nih.gov/geo (accessed on 15 June 2024) under accession numbers GSE30174, GSE14786, and GSE143791. The gene expression matrix and associated clinical data for LCa are openly available in The Cancer Genome Atlas database at https://portal.gdc.cancer.gov/ (accessed on 20 August 2024). The sensitivity analysis data for multiple tumor cell lines are openly available in the DepMap database at https://depmap.org/portal/home/#/ (accessed on 15 December 2024). The RNA-seq data generated in this study have been deposited in the NCBI Gene Expression Omnibus under accession number GSE337532.

## Supplementary Materials

The following supporting information can be downloaded at: https://www.mdpi.com/article/10.3390/cells15151394/s1, Figure S1: Screening of genes associated with FIN-enhanced cuproptosis; Figure S2: FKBP5 is involved in cuproptosis induced by FINs; Figure S3: FINs promote cuproptosis through oxidative stress; Figure S4: FKBP5 facilitates cuproptosis induced by ferroptosis-inducing agents; Figure S5: FKBP5 plays a role in modulating the Nrf2/HO-1 signaling pathway; Figure S6: The Nrf2/HO-1 pathway is involved in FIN-enhanced cuproptosis sensitization; Figure S7: Additional Co-IP and Fkbp5 knockdown/ML385 rescue analyses in LCaRF cellular models; Figure S8: Quantification of key microscopy-based endpoints in LCaRF cellular and mouse models; Figure S9: Schematic representation of FIN-enhanced CIN-induced cuproptosis in the LCaRF model; Table S1: The sources of the antibodies and reagents employed in current study; Table S2: Quantitative Reverse Transcription PCR primer sequences; Table S3: Transfection of intervention sequences.
