# Supplementary material for: Ferroptosis Inducers Combined with Copper Ionophores Aggravate Lung Cancer-Related Fatigue via GSH Depletion and FKBP5-Associated Impairment of Nrf2/HO-1 Signaling
Source: Cells. 2026 Jul 31;15(15):1394. doi: 10.3390/cells15151394 (PMC13465117; doi:10.3390/cells15151394)
Supplement: Supplementary file 1 [file cells-15-01394-s001.zip › Table S3.pdf]

Table S3 Transfection of intervention sequences.

Plasmid name	Oligos sequences (sense 5'-3')	Oligos sequences (antisense 5'-3')
siNC	CACCGGCACTACCAGAGCTAACTCA	AAACTGAGTTAGCTCTGGTAGTGCC
siFkbp5	CAGGCGUUAUCCGUAGAAUTT	AUUCUACGGAUAACGCCUGTT
siCox5b	CACCGTCCTAAAGATAACTCGACAC	AAACGTGTCGAGTTATCTTTAGGAC
siFdx1	CACCGGCAGGCCGCTGGATCCAGCG	CACCGTGATTCTCTGCTAGATGTTG
siLias	CACCGCAGGAGTAAGACACTCCACA	AAACTGTGGAGTGTCTTACTCCTGC
