# Supplementary material for: Ferroptosis Inducers Combined with Copper Ionophores Aggravate Lung Cancer-Related Fatigue via GSH Depletion and FKBP5-Associated Impairment of Nrf2/HO-1 Signaling
Source: Cells. 2026 Jul 31;15(15):1394. doi: 10.3390/cells15151394 (PMC13465117; doi:10.3390/cells15151394)
Supplement: Supplementary file 1 [file cells-15-01394-s001.zip › Table S2.pdf]

Table S2 Quantitative Reverse Transcription PCR primer sequences.

Gene	Forward	Reverse
Gapdh	5'-TCTTGGGCTACACTGAGGA-3'	5'-ATACCAGGAAATGAGCTTGA-3'
Runx3	5'-CCCAACTTCCTCTGCTCCG-3'	5'-GGTCACCACCGTTCCATCC-3'
Fkbp5	5'-GGACAGTGCCAATGAGAA-3'	5'-GATTGACTGCCAACACCT-3'
Sytl3	5'-ACCCTCCCGACCCTGTTT-3'	5'-TCCGTGTCTCAAGCCTCC-3'
Dusp2	5'-TTCTTGCGAGGCGGTTTC-3'	5'-CTCGGGTCAGAGTTGCTATTTT-3'
Ms4a7	5'-GCTTATGTCGGTGCTATG-3'	5'-TGATCTTGTGATTGAGGC-3'
Il2rb	5'-TGTCGAAGAGGAGGTGGA-3'	5'-CGGGAAGGCACAGTAGTC-3'
Lcp1	5'-TGGACCAAGATGGAAAGA-3'	5'-TACCAACGCTGGACTGCT-3'
Prf1	5'-GGGATGATGACCTTCTTGG-3'	5'-CACCCTGCCGTGGTTTAG-3'
Gzmb	5'-AGGAGAAGACCCAGCAAG-3'	5'-CAACCAGCCACATAGCAC-3'
Dlst	5'-TGGAACACCCATTATCAAC-3'	5'-AGGTCAGGGCTACATACATC-3'
Cox5a	5'-TCCATTCGCTGCTATTCT-3'	5-TGTATTCATCCCTTTACGC-3'
Cox5b	5'-TGGCTTCTGGAGGTGGTG-3'	5'-GTTGCTGATGGACGGGAC-3'
Ankrd9	5'-CTGCTAGGAGACCAGCTACGC-3'	5'-GGCACGGACAAAGAGGGAG-3'
Gls	5'-AGGGTGAAGTCGGTGATA-3'	5'-ATGGCTGACAGAGCAAAC-3'
Lipt1	5'-CCGTACTGCGGGATAAAG-3'	5'-TCCAGGGAACATCGTCTC-3'
Pdhb	5'-GCCACAATTTGGAGTAGGA-3'	5'-AGGCATAGGGACATCAGC-3'
Atp7b	5'-GGCTCAGATTGTAAAGTTGG-3'	5'-TTGCTAGGGCTAGGAAAGTA-3'
Atp7a	5'-TTTGATACACCTCCTATGC-3'	5'-TAGTGGCTTCAGTTGCTT-3'
Slc31a1	5'-TCATCCAGGTAGTCATCAGC-3'	5'-GAGAAAGTATCCCGTCCC-3'
Fdx1	5'-CGAGACGCTAACGACCAA-3'	5'-CAAGTAGAGCAAGCCAACG-3'
Lias	5'-GGTGAGACAGACGAGCAA-3'	5'-AATCCAAGTTCATTCCCTA-3'
Dld	5'-CCTTTACTCAGAATTTGGGACT-3'	5'-GACCAGCGACCACATCTC-3'
Dlat	5'-GAAAGTTCCCGAAGCAAA-3'	5'-TGGAGGCTAAAGAAACAAC-3'
Pdha1	5'-AATCATTCGTGGTTTCTGT-3'	5'-GTGGTCCGTAGGGTTTAT-3'
Mtf1	5'-CCAGGACTTTCTGTTGTTG-3'	5'-GATGGTTATGGTGGGATTA-3'
Dbt	5'-TTCGGGTATTGTGATGAG-3'	5'-GAAGCCTTGTAGGTGATGT-3'
Pold1	5'-GATGCCAACGCCAAGGTA-3'	5'-GGATGGGTGATGGGAAGTG-3'
Cox4i1	5'-CGCTCGTTCTGATTTGGG-3'	5'-CCAGCATTCGCTTGGTCT-3'
Cox4i2	5'-AGTGATGGGCTGCGTCTT-3'	5'-CGTTCTTCCGTCAGGGTG-3'
Aco2	5'-GGCAAAGCCAACTCTGTA-3'	5'-CCATGTTTCTTGTAGTAGCG-3'
Atox1	5'-GGGAGGAGTGGAGTTCAA-3'	5'-GTTGCCAGCAGGGTGTCT-3'
Ccs	5'-GGTGGTCCGCTTCCTACA-3'	5'-GGTGCCGATCAGTGTCCT-3'
Cutc	5'-GAGGTGATGAAGGCTGAC-3'	5'-AAAGGCTCTGTGGAAAGT-3'
Nfe2l2	5'-TACAGTCCCAGCAGAGTGAT-3'	5'-TCAGAAACCTCCTTCCAAA-3'
Hmox1	5'-ACAGATGGCGTCACTTCG-3'	5'-TGAGGACCCACTGGAGGA-3'
Nqo1	5'-CCTGAGCCCAGATATTGT-3'	5'-AAAGGACCGTTGTCGTAC-3'
Keap1	5'-GGTCGCCCTGTGCCTCTAT-3'	5-ACGCCAATCCTCCGTGTC-3'
