# Supplementary material for: Ferroptosis Inducers Combined with Copper Ionophores Aggravate Lung Cancer-Related Fatigue via GSH Depletion and FKBP5-Associated Impairment of Nrf2/HO-1 Signaling
Source: Cells. 2026 Jul 31;15(15):1394. doi: 10.3390/cells15151394 (PMC13465117; doi:10.3390/cells15151394)
Supplement: Supplementary file 1 [file cells-15-01394-s001.zip › Table S1.pdf]

Table S1 The sources of the antibodies and reagents employed in current study.

Reagent or resource	Source	Identifier
**Antibodies**		
GAPDH	Abclonal	Cat#A19056
FDX1	Proteintech	Cat#12592-1-AP
DLAT	Proteintech	Cat#13426-1-AP
Lipoic acid	Sigma-Aldrich	Cat#437695
FKBP5	Abcam	Cat#ab79844
COX5B	Proteintech	Cat#11418-2-AP
Nrf2	Proteintech	Cat#16396-1-AP
HO-1	Proteintech	Cat#10701-1-AP
Keap1	Proteintech	Cat#10503-2-AP
Histone H3	Proteintech	Cat#17168-1-AP
**Chemicals**		
Elesclomol (Els)	MedChemExpress	Cat#HY-12040
L-Glutathione (GSH)	MedChemExpress	Cat#HY-D0187
Sorafenib (Sora)	Selleckchem	Cat#S7397
Erastin (Era)	MedChemExpress	Cat#HY-15763
Ammonium tetrathiomolybdate (TTM)	Macklin	Cat#A828261
Tetraethylenepentamine (TEPA)	Macklin	Cat#T818822
Sodium diethyldithiocarbamate (DDC)	MedChemExpress	Cat#HY-B1637A
Tertiary butylhydroquinone (TBHQ)	MedChemExpress	Cat#HY-100489
ML385	MedChemExpress	Cat#HY-100523
Ferrostatin-1 (Fer-1)	Selleckchem	Cat#S7243
Necrostatin 2 racemate (Nec-1s)	Selleckchem	Cat#S8641
Z-VAD-FMK	Selleckchem	Cat#S7023
TRIzol Universal Reagent	Invitrogen	Cat#15596018CN
Lipofectamine™ 3000	ThermoFisher Scientific	Cat#L3000150
BODIPY™ 581/591 C11	ThermoFisher Scientific	Cat#D3861
XenoLight D-Luciferin Potassium Salt	Revvity(Perkin Elmer)	Cat#122799
L-Buthionine-(S,R)-sulfoximine (BSO)	MedChemExpress	Cat#HY-106376A
**Critical commercial assays**		
PrimeScript™ RT Reagent Kit	Yeasen	Cat#11141ES60
2X Universal SYBR Green Fast qPCR Mix	Abclonal	Cat#RK21203
RIPA lysis buffer	ThermoFisher Scientific	Cat#89901
Enhanced BCA Protein Assay Kit	Beyotime	Cat#P0010S
GSH and GSSG Assay Kit	Beyotime	Cat#S0053
Hoechst 33342/PI double-staining reagent	EallBio	Cat#02.10553
Cell Counting Kit-8	Servebio	Cat#G4103

Micro Mitochondrial complex IV Activity Assay Kit	Abbkine	Cat#KTB1880
MitoTracker Red CMXRos	Meilunbio	Cat#MB6046
Reactive Oxygen Species Assay Kit	Biosharp	Cat#BL714A
Copper Assay Kit	Sigma-Aldrich	Cat#MAK127
Ribo-Zero™ Magnetic Kit (Human/Mouse Rat)	Epicentre	Cat#MRZB12424
